# Supplementary figures and images for: Quantitative cytokine level of TNF-α, IFN-γ, IL-10, TGF-β and circulating Epstein-Barr virus DNA load in individuals with acute Malaria due to P. falciparum or P. vivax or double infection in a Malaria endemic region in Indonesia
Source: PLoS One. 2021 Dec 28;16(12):e0261923. doi: 10.1371/journal.pone.0261923 (PMC8714090; doi:10.1371/journal.pone.0261923)

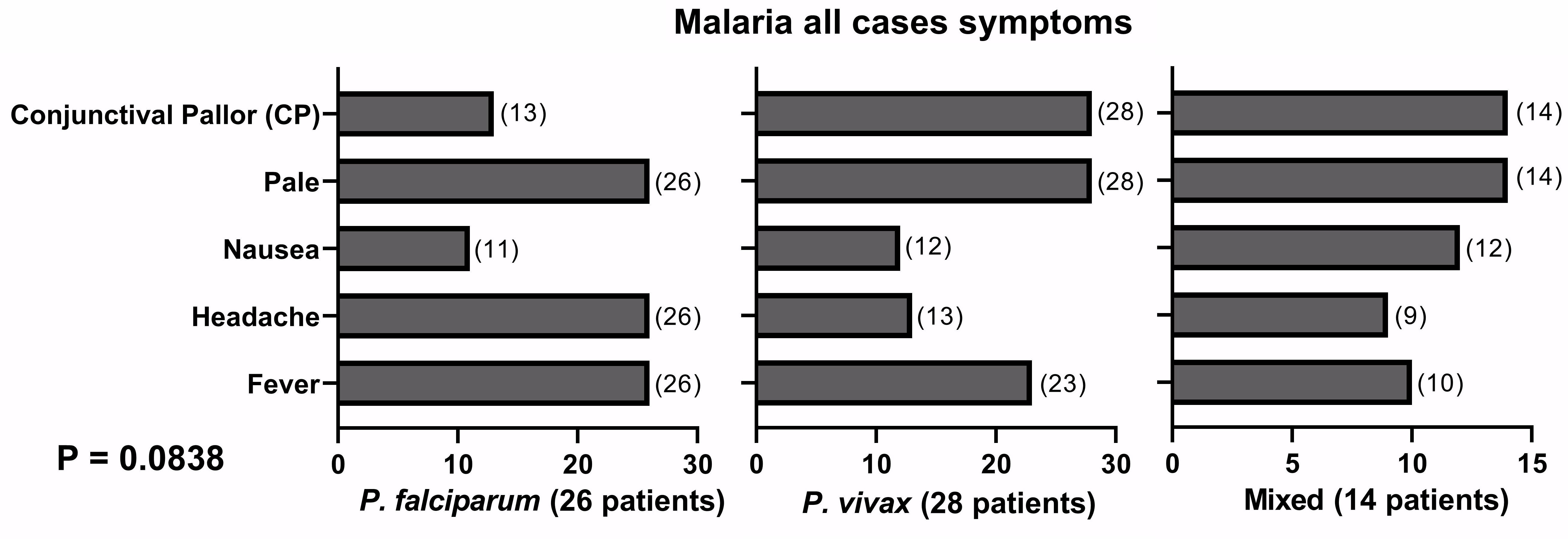

Supplement: S1 Fig — (TIF) [file pone.0261923.s001.tif]

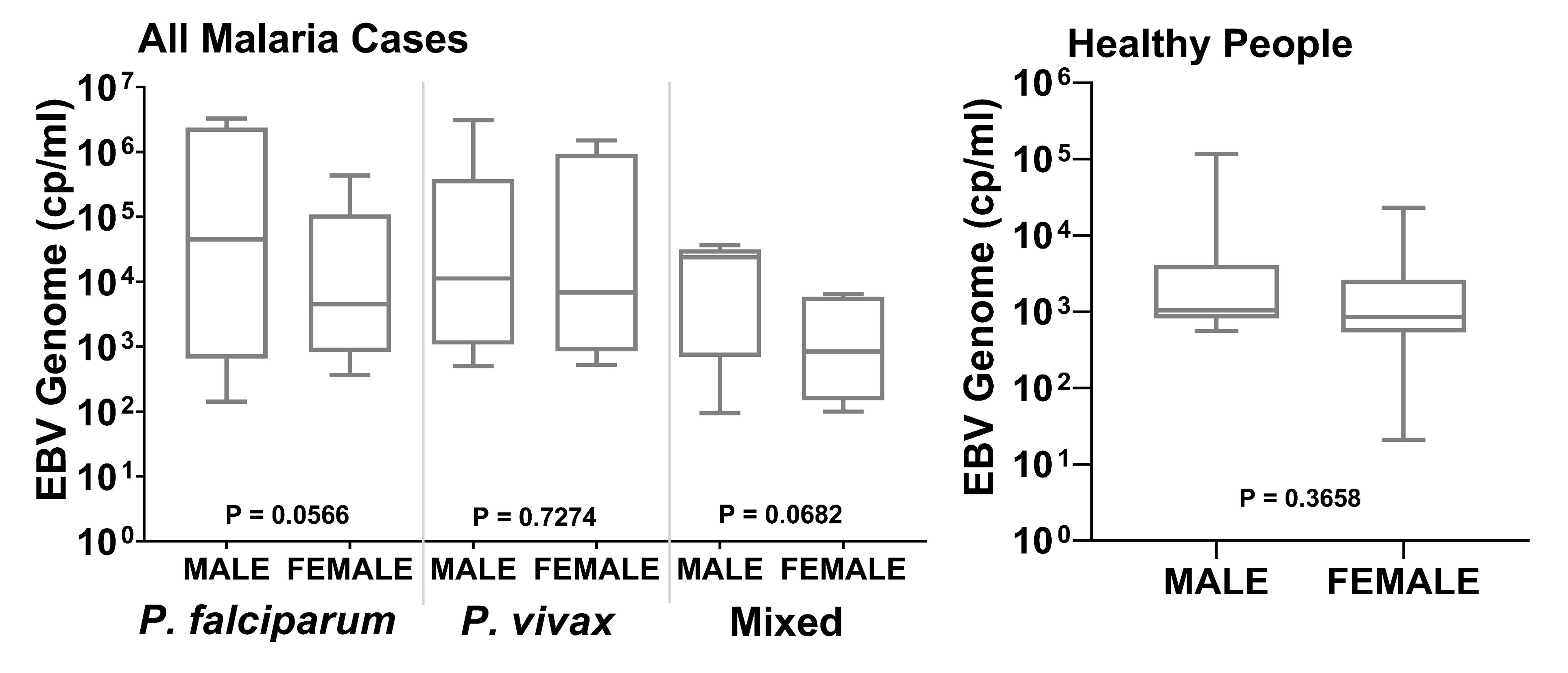

Supplement: S2 Fig — Box-plot shows 95% confidence interval and median levels and differences between male and female groups were defined by One-Way ANOVA. (TIF) [file pone.0261923.s002.tif]

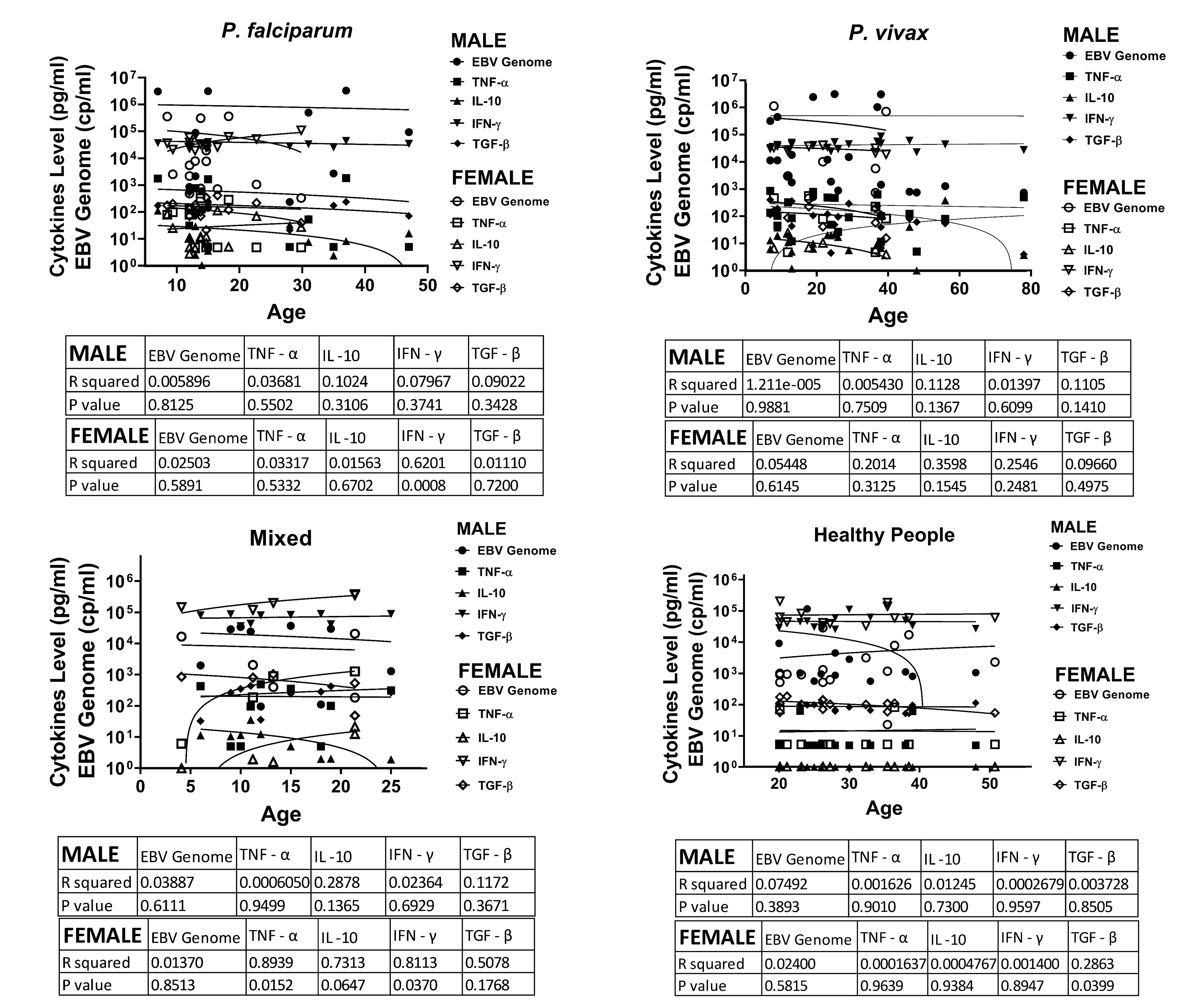

Supplement: S4 Fig — Statistical analysis was done by linear regression. (TIF) [file pone.0261923.s004.tif]
