# Supplementary material for: Quantitative cytokine level of TNF-α, IFN-γ, IL-10, TGF-β and circulating Epstein-Barr virus DNA load in individuals with acute Malaria due to P. falciparum or P. vivax or double infection in a Malaria endemic region in Indonesia
Source: PLoS One. 2021 Dec 28;16(12):e0261923. doi: 10.1371/journal.pone.0261923 (PMC8714090; doi:10.1371/journal.pone.0261923)
Supplement: Attachment — Submitted filename: Reply to reviewers comments PONE-D-21-19672 .docx [file pone.0261923.s005.docx]

**Reply to the reviewer’s comments on manuscript PONE-D-21-19672**

We are grateful to the positive and supportive comments of the reviewers.

Our answers to the reviewer’s questions are detailed here below and we have adjusted the revised manuscript accordingly.

**Reviewer #1:** We thank the reviewer for indicating the positive overall impression.
As suggested, we shortened the introduction by reducing (too many) details on inflammatory cytokines and focus better on the hypothesis / objectives of the study.
We made a statement in the results section that the distribution of EBV-DNA load and 4 individual cytokine levels was analysed for age/sex influences in patients and controls. Data on EBV-DNA male/female comparison are shown in Supplemental. Figure 2 (Box-plot with One-Way ANOVA analysis for DNA-load versus gender in each group).
Data on linear regression analysis for each parameter with age is shown in Suppl. Figure 3 (DNA loads in all Malaria cases) and Supplemental Figure 4 (EBV-DNA and 4 cytokines versus age in *P. falciparum*, *P. vivax* and Mixed infections and controls, each separately analysed for male/female status). These analyses did not influence the original results or conclusions.

#1: We thank the reviewer for this good suggestion and have emphasized the association between EBV and TNF-alpha as being specific to *P. falciparum* everywhere in the manuscript (abstract, results and discussion).

#2: We have included the correlations between EBV and TNF-alpha for the individual groups in the text of result section as suggested.

#3: The reported correlation specific to *P. falciparum* is linear and significant (P=0.8915) and holds in both male and female Malaria cases. Due to a systematic error in our DNA load calculation (see reply to reviewer 20 the EBV-DNA loads are now 6-fold lower that initially reported (modified Figure 5).

\# Discussion: We have significantly (500 words) reduced the discussion section by removing detailed background information on role(s) of cytokines in anti-Malaria defenses and likewise reduced the number of references.

**Reviewer #2:** We thank the reviewer for the positive remarks on the novelty of our findings. We have addressed the concerns on EBV-load measurement.

#1. The EBV-DNA PCR used in this study is an UNG-controlled quantitative PCR with internal calibration and H2O control for each PCR run. Erroneous amplification or PCR cross-contamination is eliminated by the UNG-procedure. Therefore, we consider it highly unlikely that the data are false-positive. This is indicated in the method section.
Unfortunately, due to the Coronavirus epidemic, the Indonesian government has officially called all research PCR instruments in the country to be used for Covid-19 testing only, and we cannot independently re-test our samples. Despite several requests and due to government blocking of new PCR kit import, we are unable to do independent PCR analysis.
By re-analysing our raw data, we found a systematic error in the EBV-DNA load calculation for all samples (the 10ul PCR input was not re-calculated properly to the 60ul eluate from the Qiagen column). This has resulted in a 6-fold reduction of the reported EBV-DNA levels. All data and figures were corrected for this systematic error.

Yet the resulting EBV-DNA levels remain rather high for healthy individuals, but now they are in line with previously reported data for healthy adults and children in malaria-risk regions in Kenia (ref. 35; Moormann et al., J. Infect. Dis. 2005). One healthy donor had near negative values and her husband had approx. 1100 copies/ml plasma. He has a known IM history > 10 years ago, with persistent high EBV-DNA load in multiple independent PCR analyses but remains without any symptoms.
Indeed, we were also surprised by elevated EBV-DNA loads found in the nine healthy controls. However, we consider this to be a reflection of the local living conditions, which may be similar to sub-Saharan Africa where healthy children and adults also have higher EBV-DNA loads (ref 35 added). Similar strenuous life conditions in Surabaya, East-Java may affect EBV homeostasis (ref 17, 18, 38). We also noted a recent publication on frequent (9.4%) subclinical Malaria infections (detected by PCR) in the Surabaya urban population (East-Java, Indonesia), where we collected our apparent healthy controls and added this as ref.26. Subclinical malaria may explain -in part- the elevated EBV-DNA levels, similar to malaria-endemic regions in Africa.

We present these arguments (see discussion) about environmental and population stressors which are especially abundant in Surabaya, East-Java compared to other parts of Indonesia (ref 17, 26, 38), and suggest that these may be responsible for the high EBV-DNA loads in this East-Java population, compared to our prior findings in Jakarta and Yogyakarta controls (non-endemic cities in West and central Java). In African healthy controls (adults and children in Kisumu-region, Kenia) the reported EBV-DNA loads are higher in a significant part of the controls compared to US healthy controls (ref. 35) and similar life-stress factors may be due to this.

Although we did not pursue the DNAse treatment, we consider that “overall” the EBV-DNA load in Surabaya EBV carriers is high, whatever the origin (cellular, apoptotic or virion) and is comparable with EBV-DNA loads in healthy African children in the Moormann 2005 study. The environmental factors that may lead to higher EBV-DNA loads (as indicated above), may influence both latent B-cell proliferation (malaria, butyrates, etc) and lytic reactivation (nitrosamines, stress hormones). It is not the aim of this study to analyse the origin of EBV-DNA in detail.

#2. We appreciate the reviewer’s comment and studied the Moormann et al., JID2005 paper. We noticed that this paper deals with children with Malaria and used local non-Malaria children and adult as controls, all being healthy and living locally in a high malaria risk region (Kisumu). Of relevance is their finding that the healthy African children displayed a wide range of EBV-DNA loads and healthy African adults in Kisumu also have elevated EBV-DNA loads compared to US-based individuals. (See our reply here above).
We eliminated the remark that “our study is ‘first’ to measure EBV loads in adults with acute malaria”.

#3. We appreciate the reviewer’s point of view. Because recent papers cite and discuss earlier seminal findings, we chose to focus on recent publications relevant to our study.

#4. We have aggregated the clinical symptoms in a graphic display (Figure supplement 1) to accommodate the reviewers suggestion.

#5. Unfortunately, no prior study has addressed this “stress” issue in Indonesia, but it is well known that food and lifestyle (nitrosamines, butyrates, phorbol esters, smoking) as well as stress hormones (glucocorticoids) are inducers of EBV reactivation and contribute to elevated EBV-DNA loads (ref. 18, 37). These factors are abundant in the East-Indonesian region, as mentioned in the discussion with related references (ref.17, 26, 38).
